# Efficacy and serious adverse events profile of the adjuvanted recombinant zoster vaccine in adults with pre-existing potential immune-mediated diseases: a pooled *post hoc* analysis on two parallel randomized trials

**DOI:** 10.1093/rheumatology/keaa424

**Published:** 2020-09-10

**Authors:** Alemnew F Dagnew, Debora Rausch, Caroline Hervé, Toufik Zahaf, Myron J Levin, Anne Schuind, for the ZOE-50/70 study group

**Affiliations:** 1 GSK, Rockville, MD, USA; 2 GSK, Philadelphia, PA, USA; 3 GSK, Wavre, Belgium; 4 GSK, Rixensart, Belgium; 5 Departments of Pediatrics and Medicine, University of Colorado Anschutz Medical Campus, Aurora, CO, USA

**Keywords:** varicella-zoster virus, potential immune-mediated diseases, rheumatoid arthritis, psoriasis, spondyloarthropathy, vaccines

## Abstract

**Abstract Objective:**

In the ZOE-50 (NCT01165177) and ZOE-70 (NCT01165229) phase 3 clinical trials, the adjuvanted recombinant zoster vaccine (RZV) demonstrated ≥90% efficacy in preventing herpes zoster (HZ) in all age groups ≥50 years. Given the increased HZ risk associated with certain underlying autoimmune diseases or their treatment regimes, we conducted a *post hoc* analysis of RZV’s efficacy against HZ and safety profile [specifically, the occurrence of serious adverse events (SAEs)] in ZOE-50/70 participants who reported pre-existing potential immune-mediated diseases (pIMDs) at enrolment and were not on immunosuppressive therapies.

**Methods:**

Adults aged ≥50 (ZOE-50) and ≥70 (ZOE-70) years were randomized to receive two doses of RZV or placebo 2 months apart. In this subgroup analysis of participants with at least one pIMD at enrolment, the efficacy was calculated for two-dose recipients who did not develop confirmed HZ before 30 days post-dose 2. SAE occurrence was evaluated for all participants who received at least one dose.

**Results:**

Of the 14 645 RZV and 14 660 placebo recipients from the ZOE-50/70 studies, 983 and 960, respectively, reported at least one pre-existing pIMD at enrolment and were included in these analyses. The most frequent pre-existing conditions were psoriasis, spondyloarthropathy and RA. Efficacy against HZ was 90.5% (95% CI: 73.5, 97.5%) overall with the lowest being 84.4% (95% CI: 30.8, 98.3%) in the 70–79-year-old age group. SAEs and fatal SAEs were similar between RZV and placebo recipients.

**Conclusion:**

In ZOE-50/70 participants with pre-existing pIMDs, RZV was highly efficacious against HZ and SAE incidence was similar between RZV and placebo recipients.

**Trial registration:**

ClinicalTrials.gov, https://clinicaltrials.gov, NCT01165177 (ZOE-50), NCT01165229 (ZOE-70).

## Graphical Abstract

**Figure:**
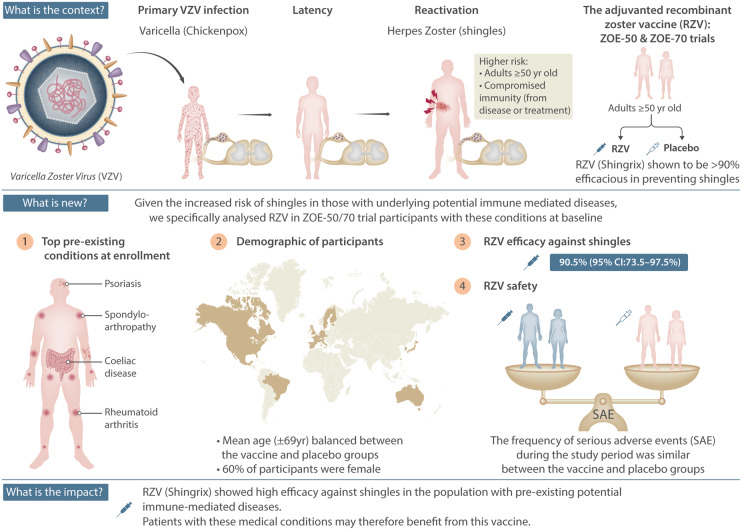

**Rheumatology key messages**

- The adjuvanted recombinant zoster vaccine is 90.5% efficacious in individuals with ≥1 potential immune-mediated disease.
- Serious adverse event incidence was similar between vaccine and placebo recipients with potential immune- mediated diseases.
- People suffering from immune-mediated diseases may benefit from vaccination with RZV.

## Introduction

Primary infection with varicella-zoster virus typically in childhood causes varicella, or chickenpox. Varicella-zoster virus establishes lifelong latency and upon its reactivation may cause herpes zoster (HZ), a typically painful, dermatomal rash, which most often occurs in older adults [1, 2]. The most common complication of HZ is postherpetic neuralgia, consisting of neuropathic pain that may persist many months after the rash resolves [3]. An adjuvanted recombinant zoster vaccine (RZV; Shingrix, GSK) was highly efficacious against both HZ and postherpetic neuralgia in two pivotal phase 3 randomized controlled trials (ZOE-50 and ZOE-70) [4, 5], and is currently licensed in several regions worldwide for use in adults aged ≥50 years.

Individuals with underlying autoimmune diseases such as RA and systemic lupus erythematosus are known to be at increased risk for developing HZ and postherpetic neuralgia [2, 6, 7]. In addition to the underlying autoimmune disease, treatment modalities for these conditions also confer an increased risk of infections and complications, including HZ. For example, corticosteroids and Janus kinase inhibitors, used in the treatment of RA and other autoimmune conditions [8, 9], are known to contribute significantly to the increased HZ incidence in this population [10–18]. Thus, individuals with autoimmune diseases may benefit from vaccination against HZ.

In the ZOE-50/70 studies, adults aged ≥50/70 years were eligible for participation if they were not considered immunocompromised by disease or treatment at enrolment and fulfilled all other inclusion criteria. The inclusion/exclusion criteria allowed for enrolment of a broad population aged ≥50 years, including individuals with potential immune-mediated diseases (pIMDs) [4, 5], which included autoimmune diseases and other inflammatory and/or neurological disorders of interest that may or may not have an autoimmune aetiology [19]. A total of 29 305 adults aged ≥50 years received at least one dose of RZV or placebo and were included in the pooled safety analyses. Pre-existing pIMDs at enrolment were reported by 983 (6.7%) RZV and 960 (6.5%) placebo recipients. In this subset of participants, most [940 (95.6%) in the RZV and 912 (95.0%) in the placebo group] did not experience a possible exacerbation of a pre-existing disease or a new onset of a different pIMD during the study period [20].

Here we present a *post hoc* analysis performed to evaluate RZV efficacy in preventing HZ and occurrence of serious adverse events (SAEs) in the pooled ZOE-50/70 population with pre-existing pIMDs.

## Methods

The ZOE-50 and ZOE-70 trials (ZOE-50/70) were large phase 3 clinical trials evaluating the efficacy and safety of RZV, conducted in parallel at the same centres in Asia, Australia, Europe, North America and Latin America in adults aged ≥50 and ≥70 years, respectively. The studies were designed to allow analyses of pooled vaccine efficacy and safety data. Protocol summaries are available at http://www.gsk-clinicalstudyregister.com (study IDs 110390 and 113077). Anonymized individual participant data and study documents can be requested for further research at www.clinicalstudydatarequest.com (study IDs 110390 and 113077).

Individuals aged ≥50 years were eligible for participation unless they had a history of HZ, were previously vaccinated against varicella or HZ, or were considered immunocompromised by underlying disease or immunosuppressive treatments. More specifically, individuals who had received immunosuppressants or immune-modifying drugs for >15 consecutive days within 6 months prior to the first vaccine dose were excluded from participation in the ZOE-50/70 studies. Prednisone <20 mg/day, or equivalent, and inhaled/topical steroid use was allowed during the study. Detailed inclusion and exclusion criteria have been described elsewhere [4, 5]. As prednisone/prednisolone is the mainstay treatment for polymyalgia rheumatica [21], the average daily dose was calculated for ZOE-50/70 participants with polymyalgia rheumatica who received prednisone/prednisolone.

At enrolment, medical conditions for each ZOE-50/70 participant were retrieved from individual medical records and/or by interview, and were recorded in participants’ medical history. Participants with at least one pIMD at enrolment were identified by searching in the clinical database with a customized Medical Dictionary for Regulatory Activities (MedDRA) query for pIMDs reported in their medical history [19]. Medical conditions that were to be identified as pIMDs by this query are provided in Supplementary Table S1, available at *Rheumatology* online.

ZOE-50/70 participants were randomized 1:1 to receive two doses of either RZV or placebo 2 months apart and followed up for safety and the occurrence of HZ. Suspected HZ cases were confirmed by either polymerase chain reaction or unanimous agreement among the five members of a HZ ascertainment committee based on available clinical information [4, 5]. Vaccine efficacy in preventing HZ was defined as 1 minus the ratio of HZ incidences (RZV over Placebo), multiplied by 100.

The analysis of vaccine efficacy in the subset of pooled ZOE-50/70 participants with pre-existing pIMDs at enrolment [overall and by age group (50–59, 60–69, 70–79 and ≥80 years)] was performed in the modified total vaccinated cohort, which included all participants who received both doses of study vaccine and did not develop a confirmed HZ episode before 30 days post-dose 2. This allowed participants to achieve full protection resulting from a two-dose schedule. For the efficacy assessment, only the first confirmed HZ episode in a participant was considered, and the follow-up period for the efficacy assessment ceased at the time of the first occurrence. Safety was assessed in the TVC, which included participants who received at least one dose: SAEs were recorded from RZV or placebo dose 1 through 12 months post-last dose, and fatal SAEs were recorded during the entire study period (median follow-up duration of 4.4 years in the TVC [20]).

All statistical analyses were performed using the SAS Drug Development software package (SAS Institute Inc., Cary, NC, USA).

## Results

### Study population results

**Fig. 1:**
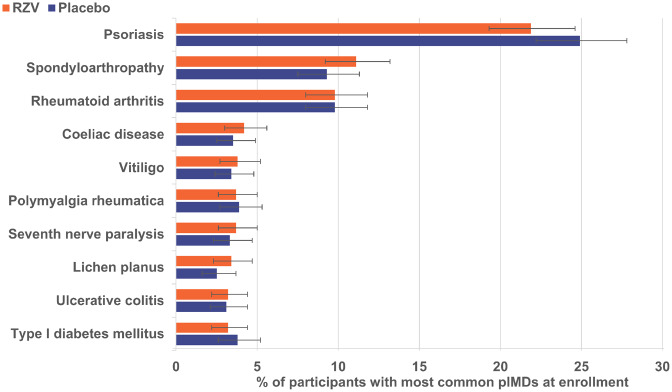
Ten most common pIMDs in both groups at enrolment (TVC) Participants with pre-existing pIMDs were identified by querying the global medical history of the participants included in TVC with a customized Medical Dictionary for Regulatory Activities query for pIMDs [19]. Error bars represent 95% CI. pIMD: potential immune-mediated disease; Placebo: participants receiving placebo; RZV: participants receiving the adjuvanted recombinant zoster vaccine; TVC: total vaccinated cohort.

From 14 645 RZV and 14 660 placebo recipients included in the pooled TVC of the ZOE-50/70 studies, 983 and 960, respectively, reported at least one pIMD at enrolment [20]. The mean age of participants having at least one pIMD at enrolment was 68.8 and 69.4 years in the RZV and placebo groups, respectively, and more than half (RZV: 59.9%, Placebo: 60.8%) were female (Table 1). The most frequent pre-existing pIMDs reported at enrolment were psoriasis, spondyloarthropathy, RA and coeliac disease; their prevalence was balanced between study groups (Fig. 1 and Supplementary Table S2, available at *Rheumatology* online). For the period ranging from 6 months before first study vaccine administration up to study end, prednisone/prednisolone use was reported by 27 out of 36 (75.0%) and 23 out of 37 (62.2%) polymyalgia rheumatica patients from the RZV and placebo groups, with average doses of 13 and 9 mg/day, respectively.

**Table 1:** Demographic characteristics of pooled ZOE-50/70 participants with pre-existing pIMDs (TVC)

Characteristic	RZV (*n* = 983)	Placebo (*n* = 960)
Age at first dose, mean (s.d.), years	68.8 (9.6)	69.4 (9.5)
Age category, *n* (%)		
50–59 years	230 (23.4)	208 (21.7)
60–69 years	165 (16.8)	156 (16.3)
70–79 years	450 (45.8)	468 (48.7)
≥80 years	138 (14.0)	128 (13.3)
Sex, *n* (%)		
Female	589 (59.9)	584 (60.8)
Male	394 (40.1)	376 (39.2)
Geographic ancestry, *n* (%)		
White – Caucasian/European	830 (84.4)	829 (86.4)
Asian – East Asian	64 (6.5)	50 (5.2)
Asian – Japanese	30 (3.1)	27 (2.8)
African/African American	11 (1.1)	7 (0.7)
White – Arabic/North African	9 (0.9)	6 (0.6)
Other	39 (4.0)	41 (4.3)

*n*: number of participants included in each group; *n* (%): number (percentage) of participants in a category; pIMD: potential immune-mediated disease; Placebo: participants receiving placebo; RZV: participants receiving the adjuvanted recombinant zoster vaccine; TVC: total vaccinated cohort; ZOE-50/70: RZV efficacy studies in adults ≥50 and ≥70 years of age, respectively.

### Efficacy in ZOE-50/70 participants with at least one pIMD at enrolment

Vaccine efficacy against HZ in pooled ZOE-50/70 participants with at least one pIMD at enrolment was 90.5% (95% CI: 73.5, 97.5%). Point estimates for efficacy were ≥84.4% across age groups (Table 2).

**Table 2:** Vaccine efficacy against HZ in pooled ZOE 50/70 participants with pre-existing pIMDs (mTVC)

	RZV				Placebo				Vaccine efficacy^b^, % (95% CI)
	*n*	No. of confirmed HZ cases	Sum of follow-up years^a^	Incidence per 1000 person-years	*n*	No. of confirmed HZ cases	Sum of follow-up years^a^	Incidence per 1000 person-years	
Overall	936	4	3611.7	1.1	923	38	3408.8	11.1	90.5 (73.5, 97.5)
50–59 YOA	222	1	885.6	1.1	201	11	775.6	14.2	92.8 (50.5, 99.8)
60–69 YOA	159	0	638.3	0.0	151	8	588.8	13.6	100 (54.9, 100)
70–79 YOA	427	2	1623.0	1.2	450	13	1647.3	7.9	84.4 (30.8, 98.3)
≥80 YOA	128	1	464.8	2.2	121	6	397.0	15.1	86.2 (-13.5, 99.7)

Vaccine efficacy was estimated against first or only HZ episode occurring during the entire study period. All efficacy estimates were adjusted by region; overall efficacy estimates were also adjusted by age strata.

a The follow-up period ceased when the first confirmed HZ episode occurred.

b Calculated with the Poisson method. HZ: herpes zoster; mTVC: modified total vaccinated cohort; *n*: number of participants included in each group; pIMD: potential immune-mediated disease; Placebo: participants receiving placebo; RZV: participants receiving the adjuvanted recombinant zoster vaccine; YOA: years of age; ZOE-50/70: RZV efficacy studies in adults ≥50 YOA and ≥70 YOA, respectively.

### SAEs in ZOE-50/70 participants with at least one pIMD at enrolment

**Fig. 2:**
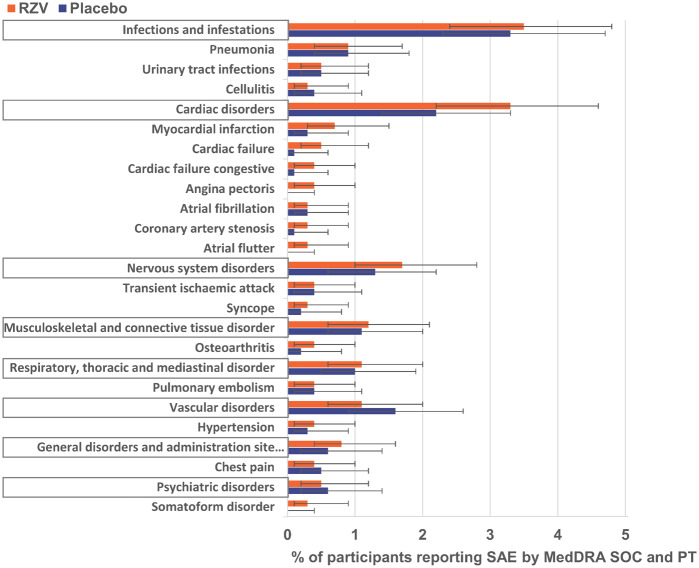
SAEs in participants with pre-existing pIMDs reported through 365 days post-last vaccine dose (TVC) SAEs by MedDRA SOC (framed) and PT are presented here. Per PT, only SAEs reported by ≥0.3% of RZV recipients are presented here. Error bars represent 95% CI. MedDRA: Medical Dictionary for Regulatory Activities; pIMD: potential immune-mediated disease; Placebo: participants receiving placebo; PT: preferred term; RZV: participants receiving the adjuvanted recombinant zoster vaccine; SAE: serious adverse event; SOC: system organ class; TVC: total vaccinated cohort; YOA: years of age.

From first dose through 365 days post-last dose, at least one SAE was reported by 144 (14.6%) RZV and 112 (11.7%) placebo recipients with at least one pre-existing pIMD (Supplementary Table S3, available at *Rheumatology* online). The most frequently reported SAEs by MedDRA system organ class were infections and infestations, followed by cardiac disorders. The most frequently reported SAEs in both treatment groups by MedDRA preferred term (PT) were pneumonia, myocardial infarction, urinary tract infection and cardiac failure (Fig. 2 and Supplementary Table S3, available at *Rheumatology* online).

From first dose through 365 days post-last dose, fatal SAEs were reported for 12 (1.2%) RZV and 9 (0.9%) placebo recipients with at least one pre-existing pIMD (Supplementary Table S3, available at *Rheumatology* online). In the RZV group, the following 12 SAEs with fatal outcome by MedDRA PT were recorded: acute myocardial infarction, myocardial infarction, large intestinal obstruction, sudden death, neutropenic sepsis, pneumonia, skull fracture, ovarian cancer, prostate cancer, rectal adenocarcinoma, cerebrovascular accident, pneumonia aspiration and two pancreatic carcinomas. In the placebo group, the nine SAEs with fatal outcome recorded were: acute myocardial infarction, chronic hepatic failure, large cell lung cancer, metastases to central nervous system, cerebrovascular accident, azotaemia, pulmonary fibrosis and two pancreatic carcinomas. There was no pattern regarding the causes of death for these participants and these were independent from underlying pIMDs (Supplementary Table S1, available at *Rheumatology* online) and expected for an elderly population.

From first vaccination through study end, fatal SAEs were reported for 50 (5.1%) RZV and 63 (6.6%) placebo recipients (Supplementary Table S3, available at *Rheumatology* online). The most frequent of these by MedDRA PT were pneumonia, cardiac failure and lung neoplasm malignant (Fig. 3 and Supplementary Table S3, available at *Rheumatology* online).

**Figure:**
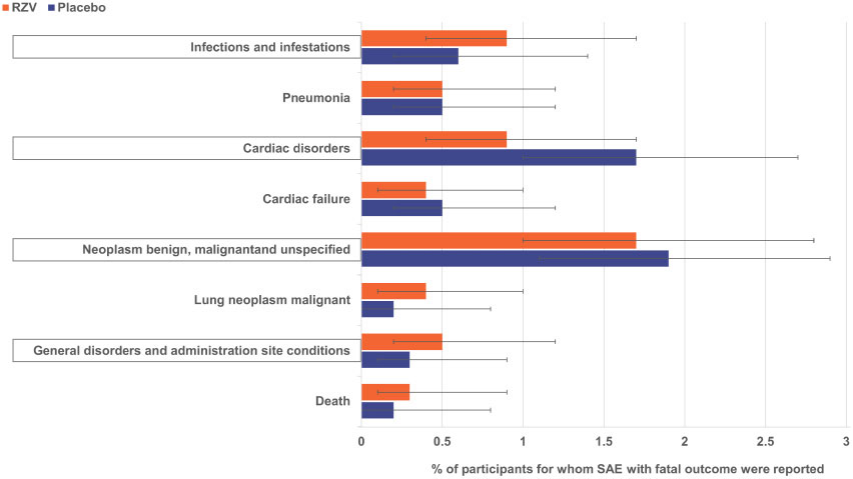
**Fig. 3 **SAEs with fatal outcome in participants with pre-existing pIMDs reported during the entire study period (TVC) SAEs by MedDRA SOC (framed) and PT are presented here. Per PT, only SAEs reported by ≥0.3% of RZV recipients are presented here. Error bars represent 95% CI. MedDRA: Medical Dictionary for Regulatory Activities; pIMD: potential immune-mediated disease; Placebo: participants receiving placebo; PT: preferred term; RZV: participants receiving the adjuvanted recombinant zoster vaccine; SAE: serious adverse event; SOC: system organ class; TVC: total vaccinated cohort.

## Discussion

This *post hoc* analysis showed that RZV is highly efficacious in the prevention of HZ and did not identify any imbalance between the vaccine and the placebo group in terms of SAEs in adults ≥50 years of age enrolled in the ZOE-50/70 studies who had at least one pre-existing pIMD at study entry.

The ZOE-50 study showed that RZV is 97.2% (95% CI 93.7, 99.0%) efficacious in preventing HZ in adults aged ≥50 years [4]. Pooled ZOE-50/70 data show that efficacy is maintained at >90% even in the oldest age group (≥80 year-olds) [5], and is not impacted by geographic ancestry/ethnicity, region, gender [22] or comorbidities [23]. The individuals included in our *post hoc* analysis were similar in terms of demographic characteristics (Table 1) with population in pooled ZOE-50/70, where the mean age of the participants was 68.6 years; 58.2% were female and most of the participants (73.7%) were white [20]. Our analysis shows that efficacy of RZV is also high in individuals having pre-existing pIMDs when enrolled in the ZOE-50/70 studies, underscoring the robustness of RZV in the prevention of HZ. It should, however, be noted that per inclusion/exclusion criteria, study participants were not considered immunocompromised by disease or treatment at enrolment. This includes individuals who received prednisone doses <20 mg/day (e.g. polymyalgia rheumatica patients), which is a rather permissive threshold, given that, more conservatively, doses >10 mg/day are already accounted for as immunosuppressive [24].

In this subset of pooled ZOE-50/70 participants with pre-existing pIMDs at enrolment, the occurrence of SAEs within 1 year post-last study vaccine dose was similar between the RZV [14.6% (95% CI: 12.5, 17.0%)] and the placebo [11.7% (95% CI: 9.7, 13.9%)] groups, and, in the RZV group, it appeared to be higher than in the overall pooled ZOE-50/70 population [RZV: 10.1% (95% CI: 9.6, 10.6%), placebo: 10.4% (95% CI: 9.9, 10.9%)] [20]. In the overall ZOE-50/70 population, the most frequently reported SAEs by MedDRA system organ class were infections and infestations, followed by cardiac disorders [20]. In the subset of participants reporting pIMDs at enrolment, infections and infestations and cardiac disorders were also the most frequently reported SAEs by system organ class. By MedDRA PT, the most frequently reported SAE was pneumonia both in the overall study population [20] and in the subset of participants having pIMDs at enrolment. In the subset of participants reporting pIMDs at enrolment, more RZV than placebo recipients: 32 [3.3% (95% CI: 2.2, 4.6%)] *vs* 21 [2.2% (95% CI: 1.4, 3.3%)] reported cardiac disorders, but the CIs for the corresponding occurrences are overlapping. No temporal clustering of SAEs was observed.

In the subset of pooled ZOE-50/70 participants with pre-existing pIMDs at enrolment, occurrence of fatal SAEs during the entire study period was similar between the RZV [5.1% (95% CI: 3.8, 6.7%)] and the placebo [6.6% (95% CI: 5.1, 8.3%)] groups and appeared to be similar to the overall pooled ZOE-50/70 population [RZV: 4.3% (95% CI: 4.0, 4.7%), placebo: 4.6% (95% CI: 4.3, 5.0%)] [20]. The reported SAEs in this population were generally consistent with events anticipated to occur in this age group, and the overall SAE profile observed is similar to that observed in the full study population [20].

Occurrence of exacerbations and new onset pIMDs in the subset of pooled ZOE-50/70 participants with pre-existing pIMDs at enrolment has been described previously [20]. Briefly, 27 (2.8%) participants in the RZV group and 27 (2.8%) participants in placebo group reported a possible exacerbation of a pre-existing pIMD and 16 (1.6%) and 23 (2.4%), respectively, reported a new onset of a different pIMD during the whole study period [20]. In addition, our data show a balance between study groups in the frequency and nature of SAEs, confirming the favourable safety profile of RZV in populations with pIMDs.

The limitations of this *post hoc* analysis must be considered when interpreting its results. The presented analyses were not controlled for type 1 error. More importantly, the ZOE-50/70 studies were not powered to assess vaccine efficacy or safety in subsets such as study participants with pre-existing pIMDs. Nonetheless, the number of pooled ZOE-50/70 participants with pIMDs at enrolment was substantial and therefore allowed for the estimation of vaccine efficacy against HZ and occurrence of SAEs in this population subset, although not by discrete pre-existing pIMDs reported at enrolment. Pre-existing pIMDs at enrolment were not taken into consideration for randomization. However, the prevalence of each of the most frequent pIMDs was similar between the RZV and placebo groups. In addition, persons with pre-existing pIMDs who were undergoing immunosuppressive treatment were not enrolled in the ZOE-50/70 studies. However, RZV was also shown to be safe, immunogenic and highly efficacious against HZ in populations that are severely immunosuppressed by disease and/or its treatment [25–28].

## Conclusions

In the subset of participants from the ZOE-50/70 clinical trials who had at least one pre-existing pIMD at enrolment, RZV was 90.5% efficacious against confirmed HZ cases, and the safety profile in terms of SAE occurrence was similar in the vaccine and placebo groups.

**Trademark statement:** *Shingrix* is a trademark of the GSK group of companies.

## Supplementary Material

keaa424_Supplementary_Data
