# Supplementary material for: Efficacy and serious adverse events profile of the adjuvanted recombinant zoster vaccine in adults with pre-existing potential immune-mediated diseases: a pooled post hoc analysis on two parallel randomized trials
Source: Rheumatology (Oxford). 2020 Sep 10;60(3):1226–33. doi: 10.1093/rheumatology/keaa424 (PMC7937016; doi:10.1093/rheumatology/keaa424)
Supplement: keaa424_Supplementary_Data [file keaa424_supplementary_data.docx]

# SUPPLEMENTARY MATERIAL

# Supplementary Table S1. Pre-defined medical conditions considered as potential immune-mediated diseases in effect at time of pooled analysis

| **Neuroinflammatory disorders** | **Musculoskeletal disorders** | **Skin disorders** |
| --- | --- | --- |
| - Cranial nerve disorders, including paralyses/paresis (e.g. Bell’s palsy) - Optic neuritis - Multiple sclerosis - Transverse myelitis - Guillain-Barré syndrome, including Miller Fisher syndrome and other variants - Acute disseminated encephalomyelitis, including site specific variants: e.g. non-infectious encephalitis, encephalomyelitis, myelitis, myeloradiculoneuritis - Myasthenia gravis, including Lambert-Eaton myasthenic syndrome - Immune-mediated peripheral neuropathies and plexopathies, (including chronic inflammatory demyelinating polyneuropathy, multifocal motor neuropathy and polyneuropathies associated with monoclonal gammopathy). - Narcolepsy | - Systemic lupus erythematosus and associated conditions - Systemic Scleroderma (Systemic sclerosis), including diffuse systemic form and CREST syndrome - Idiopathic inflammatory myopathies, including Dermatomyositis, Polymyositis, - Antisynthetase syndrome - Rheumatoid arthritis and associated conditions including Juvenile chronic arthritis and Still’s disease) - Polymyalgia rheumatica - Spondyloarthritis, including ankylosing spondylitis, reactive arthritis (Reiter's Syndrome) and undifferentiated spondyloarthritis - Psoriatic arthropathy - Relapsing polychondritis - Mixed connective tissue disorder | - Psoriasis - Vitiligo - Erythema nodosum - Autoimmune bullous skin diseases (including pemphigus, pemphigoid and dermatitis herpetiformis) - Alopecia areata - Lichen planus - Sweet’s syndrome - Localised Scleroderma (Morphoea) |
| **Liver disorders** | **Gastrointestinal disorders** | **Endocrine disorders** |
| - Autoimmune hepatitis - Primary biliary cirrhosis - Primary sclerosing cholangitis - Autoimmune cholangitis. | - Inflammatory Bowel disease, including Crohn’s disease, ulcerative colitis, microscopic colitis, ulcerative proctitis - Celiac disease - Autoimmune pancreatitis | - Autoimmune thyroiditis (including Hashimoto thyroiditis) - Grave's or Basedow’s disease - Diabetes mellitus type I - Addison’s disease - Polyglandular autoimmune syndrome - Autoimmune hypophysitis |
| **Vasculitides** | **Blood disorders** | **Others** |
| - Large vessels vasculitis including: giant cell arteritis such as Takayasu's arteritis and temporal arteritis. - Medium sized and/or small vessels vasculitis including: polyarteritis nodosa, Kawasaki's disease, microscopic polyangiitis, Wegener's granulomatosis, Churg–Strauss syndrome (allergic granulomatous angiitis), Buerger’s disease (thromboangiitis obliterans), necrotizing vasculitis and anti-neutrophil cytoplasmic antibody (ANCA) positive vasculitis (type unspecified), Henoch-Schonlein purpura, Behcet's syndrome, leukocytoclastic vasculitis. | - Autoimmune hemolytic anemia - Autoimmune thrombocytopenia - Antiphospholipid syndrome - Pernicious anemia - Autoimmune aplastic anaemia - Autoimmune neutropenia - Autoimmune pancytopenia | - Autoimmune glomerulonephritis (including IgA nephropathy, glomerulonephritis rapidly progressive, membranous glomerulonephritis, membranoproliferative glomerulonephritis, and mesangioproliferative glomerulonephritis) - Ocular autoimmune diseases (including autoimmune uveitis and autoimmune retinopathy) - Autoimmune myocarditis/cardiomyopathy - Sarcoidosis - Stevens-Johnson syndrome - Sjögren’s syndrome - Idiopathic pulmonary fibrosis - Goodpasture syndrome - Raynaud’s phenomenon |

# Supplementary Table S2. Pre-existing pIMDs at enrollment in the in ZOE-50/70 studies

| **MedDRA SOC** | **MedDRA PT** | **n** | **RZV (N=983)**  **% (95% CI)** | **n** | **Placebo (N=960)**  **% (95% CI)** |
| --- | --- | --- | --- | --- | --- |
| Blood and lymphatic system disorders | Pernicious anemia | 19 | 1.9 (1.2–3.0) | 35 | 3.6 (2.6–5.0) |
|  | Immune thrombocytopenic purpura | 9 | 0.9 (0.4–1.7) | 8 | 0.8 (0.4–1.6) |
| Endocrine disorders | Autoimmune thyroiditis | 27 | 2.7 (1.8–4.0) | 33 | 3.4 (2.4–4.8) |
|  | Basedow’s disease | 19 | 1.9 (1.2–3.0) | 19 | 2.0 (1.2–3.1) |
| Gastrointestinal disorders | Coeliac disease | 41 | 4.2 (3.0–5.6) | 34 | 3.5 (2.5–4.9) |
|  | Colitis ulcerative | 31 | 3.2 (2.2–4.4) | 30 | 3.1 (2.1–4.4) |
|  | Crohn’s disease | 13 | 1.3 (0.7–2.3) | 15 | 1.6 (0.9–2.6) |
| Hepatobiliary disorders | Biliary cirrhosis primary | 5 | 0.5 (0.2–1.2) | 3 | 0.3 (0.1–0.9) |
| Immune system disorders | Sarcoidosis | 22 | 2.2 (1.4–3.4) | 16 | 1.7 (1.0–2.7) |
| Infections and infestations | Encephalitis | 3 | 0.3 (0.1–0.9) | 3 | 0.3 (0.1–0.9) |
| Metabolism and nutrition disorders | Type 1 diabetes mellitus | 31 | 3.2 (2.2–4.4) | 36 | 3.8 (2.6–5.2) |
| Musculoskeletal and connective tissue disorders | Spondyloarthropathy | 109 | 11.1 (9.2–13.2) | 89 | 9.3 (7.5–11.3) |
|  | Rheumatoid arthritis | 96 | 9.8 (8.0–11.8) | 94 | 9.8 (8.0–11.8) |
|  | Polymyalgia rheumatica | 36 | 3.7 (2.6–5.0) | 37 | 3.9 (2.7–5.3) |
|  | Sjogren’s syndrome | 23 | 2.3 (1.5–3.5) | 21 | 2.2 (1.4–3.3) |
|  | Ankylosing spondylitis | 18 | 1.8 (1.1–2.9) | 9 | 0.9 (0.4–1.8) |
|  | Psoriatic arthropathy | 9 | 0.9 (0.4–1.7) | 5 | 0.5 (0.2–1.2) |
|  | Spondylitis | 9 | 0.9 (0.4–1.7) | 15 | 1.6 (0.9–2.6) |
|  | Arthritis reactive | 6 | 0.6 (0.2–1.3) | 3 | 0.3 (0.1–0.9) |
| Nervous system disorders | VII^th^ nerve paralysis | 36 | 3.7 (2.6–5.0) | 32 | 3.3 (2.3–4.7) |
|  | Radiculitis cervical | 14 | 1.4 (0.8–2.4) | 8 | 0.8 (0.4–1.6) |
|  | Neuritis | 13 | 1.3 (0.7–2.3) | 5 | 0.5 (0.2–1.2) |
|  | Radiculitis | 13 | 1.3 (0.7–2.3) | 10 | 1.0 (0.5–1.9) |
|  | Anosmia | 6 | 0.6 (0.2–1.3) | 8 | 0.8 (0.4–1.6) |
|  | Facial paresis | 5 | 0.5 (0.2–1.2) | 10 | 1.0 (0.5–1.9) |
|  | Mononeuritis | 4 | 0.4 (0.1–1.0) | 2 | 0.2 (0.0–0.8) |
|  | Multiple sclerosis | 4 | 0.4 (0.1–1.0) | 5 | 0.5 (0.2–1.2) |
|  | Narcolepsy | 4 | 0.4 (0.1–1.0) | 0 | 0.0 (0.0–0.4) |
|  | Vocal cord paralysis | 4 | 0.4 (0.1–1.0) | 2 | 0.2 (0.0–0.8) |
| Renal and urinary disorders | IgA nephropathy | 3 | 0.3 (0.1–0.9) | 2 | 0.2 (0.0–0.8) |
| Respiratory, thoracic and mediastinal disorders | Pulmonary fibrosis | 17 | 1.7 (1.0–2.8) | 12 | 1.3 (0.6–2.2) |
| Skin and subcutaneous tissue disorders | Psoriasis | 215 | 21.9 (19.3–24.6) | 239 | 24.9 (22.2–27.8) |
|  | Vitiligo | 37 | 3.8 (2.7–5.2) | 33 | 3.4 (2.4–4.8) |
|  | Lichen planus | 33 | 3.4 (2.3–4.7) | 24 | 2.5 (1.6–3.7) |
|  | Alopecia areata | 5 | 0.5 (0.2–1.2) | 8 | 0.8 (0.4–1.6) |
|  | Erythema nodosum | 5 | 0.5 (0.2–1.2) | 4 | 0.4 (0.1–1.1) |
| Vascular disorders | Raynaud’s phenomenon | 27 | 2.7 (1.8–4.0) | 29 | 3.0 (2.0–4.3) |
|  | Arteritis | 12 | 1.2 (0.6–2.1) | 14 | 1.5 (0.8–2.4) |
|  | Temporal arteritis | 4 | 0.4 (0.1–1.0) | 4 | 0.4 (0.1–1.1) |
|  | Vasculitis | 4 | 0.4 (0.1–1.0) | 2 | 0.2 (0.0–0.8) |
|  | Thromboangiitis obliterans | 3 | 0.3 (0.1–0.9) | 1 | 0.1 (0.0–0.6) |

RZV: participants receiving the adjuvanted recombinant zoster vaccine; Placebo: participants receiving placebo; ZOE-50/70: RZV efficacy studies in adults ≥50 YOA (NCT01165177) and ≥70 YOA (NCT01165229), respectively; CI: confidence interval; pIMD: potential immune mediated disease; N: number of participants with at least 1 administered dose; n/%: number/percentage of participants reporting the condition at study enrollment; MedDRA: medical dictionary for regulatory activities; SOC: system organ class; PT: preferred term; YOA: years of age

Notes: Only pIMDs reported by ≥0.3% of RZV recipients per PT are presented here.

# Supplementary Table S3. SAEs and SAEs with fatal outcome in pooled ZOE-50/70 participants with pre-existing pIMDs (TVC)

| **MedDRA SOC and PT** | **n** | **RZV (N=983)**  **% (95% CI)** | **n** | **Placebo (N=960)**  **% (95% CI)** |
| --- | --- | --- | --- | --- |
| ***SAEs recorded through 365 days after last dose*** | **144** | **14.6 (12.5–17.0)** | **112** | **11.7 (9.7–13.9)** |
| **Infections and infestations** | *34* | *3.5 (2.4–4.8)* | *32* | *3.3 (2.3–4.7)* |
| Pneumonia | 9 | 0.9 (0.4–1.7) | 9 | 0.9 (0.4–1.8) |
| Urinary tract infections | 5 | 0.5 (0.2–1.2) | 5 | 0.5 (0.2–1.2) |
| Cellulitis | 3 | 0.3 (0.1–0.9) | 4 | 0.4 (0.1–1.1) |
| **Cardiac disorders** | *32* | *3.3 (2.2–4.6)* | *21* | *2.2 (1.4–3.3)* |
| Myocardial infarction | 7 | 0.7 (0.3–1.5) | 3 | 0.3 (0.1–0.9) |
| Cardiac failure | 5 | 0.5 (0.2–1.2) | 1 | 0.1 (0.0–0.6) |
| Cardiac failure congestive | 4 | 0.4 (0.1–1.0) | 1 | 0.1 (0.0–0.6) |
| Angina pectoris | 4 | 0.4 (0.1–1.0) | 0 | 0.0 (0.0–0.4) |
| Atrial fibrillation | 3 | 0.3 (0.1–0.9) | 3 | 0.3 (0.1–0.9) |
| Coronary artery stenosis | 3 | 0.3 (0.1–0.9) | 1 | 0.1 (0.0–0.6) |
| Atrial flutter | 3 | 0.3 (0.1–0.9) | 0 | 0.0 (0.0–0.4) |
| **Nervous system disorders** | *17* | *1.7 (1.0–2.8)* | *12* | *1.3 (0.6–2.2)* |
| Transient ischemic attack | 4 | 0.4 (0.1–1.0) | 4 | 0.4 (0.1–1.1) |
| Syncope | 3 | 0.3 (0.1–0.9) | 2 | 0.2 (0.0–0.8) |
| **Musculoskeletal and connective tissue disorders** | *12* | *1.2 (0.6–2.1)* | *11* | *1.1 (0.6–2.0)* |
| Osteoarthritis | 4 | 0.4 (0.1–1.0) | 2 | 0.2 (0.0–0.8) |
| **Respiratory, thoracic and mediastinal disorders** | *11* | *1.1 (0.6–2.0)* | *10* | *1.0 (0.5–1.9)* |
| Pulmonary embolism | 4 | 0.4 (0.1–1.0) | 4 | 0.4 (0.1–1.1) |
| **Vascular disorders** | *11* | *1.1 (0.6–2.0)* | *15* | *1.6 (0.9–2.6)* |
| Hypertension | 4 | 0.4 (0.1–1.0) | 3 | 0.3 (0.1–0.9) |
| **General disorders and administration site conditions** | *8* | *0.8 (0.4–1.6)* | *6* | *0.6 (0.2–1.4)* |
| Chest pain | 4 | 0.4 (0.1–1.0) | 5 | 0.5 (0.2–1.2) |
| **Psychiatric disorders** | *5* | *0.5 (0.2–1.2)* | *6* | *0.6 (0.2–1.4)* |
| Somatoform disorder | 3 | 0.3 (0.1–0.9) | 0 | 0.0 (0.0–0.4) |
| ***SAEs with fatal outcome*** ***recorded through 365 days after last dose*** | **12** | **1.2 (0.6–2.1)** | **9** | **0.9 (0.4–1.8)** |
| ***SAEs with fatal outcome recorded during the entire study period*** | **50** | **5.1 (3.8–6.7)** | **63** | **6.6 (5.1–8.3)** |
| **Infections and infestations** | *9* | *0.9 (0.4–1.7)* | *6* | *0.6 (0.2–1.4)* |
| Pneumonia | 5 | 0.5 (0.2–1.2) | 5 | 0.5 (0.2–1.2) |
| Sepsis | 2 | 0.2 (0.0–0.7) | 1 | 0.1 (0.0–0.6) |
| **Cardiac disorders** | *9* | *0.9 (0.4–1.7)* | *16* | *1.7 (1.0–2.7)* |
| Cardiac failure | 4 | 0.4 (0.1–1.0) | 5 | 0.5 (0.2–1.2) |
| Acute myocardial infarction | 1 | 0.1 (0.0–0.6) | 3 | 0.3 (0.1–0.9) |
| Cardiac failure congestive | 1 | 0.1 (0.0–0.6) | 2 | 0.2 (0.0–0.8) |
| Cardiac arrest | 1 | 0.1 (0.0–0.6) | 2 | 0.2 (0.0–0.8) |
| Myocardial infarction | 2 | 0.2 (0.0–0.7) | 1 | 0.1 (0.0–0.6) |
| **Neoplasms benign, malignant and unspecified (including cysts and polyps)** | *17* | *1.7 (1.0–2.8)* | *18* | *1.9 (1.1–2.9)* |
| Lung neoplasm malignant | 4 | 0.4 (0.1–1.0) | 2 | 0.2 (0.0–0.8) |
| Pancreatic carcinoma | 2 | 0.2 (0.0–0.7) | 1 | 0.2 (0.0–0.8) |
| Metastatic carcinoma of the bladder | 2 | 0.2 (0.0–0.7) | 0 | 0.0 (0.0–0.4) |
| **General disorders and administration site conditions** | *5* | *0.5 (0.2–1.2)* | *3* | *0.3 (0.1–0.9)* |
| Death | 3 | 0.3 (0.1–0.9) | 2 | 0.2 (0.0–0.8) |
| **Nervous system disorders** | 2 | 0.2 (0.0–0.7) | 4 | 0.4 (0.1–1.1) |
| Cerebrovascular accident | 1 | 0.1 (0.0–0.6) | 2 | 0.2 (0.0–0.8) |
| **Respiratory, thoracic and mediastinal disorders** | 3 | 0.3 (0.1–0.9) | 4 | 0.4 (0.1–1.1) |
| Pneumonia aspiration | 1 | 0.1 (0.0–0.6) | 3 | 0.3 (0.1–0.9) |
| Pulmonary embolism | 1 | 0.1 (0.0–0.6) | 2 | 0.2 (0.0–0.8) |
| Respiratory failure | 1 | 0.1 (0.0–0.6) | 2 | 0.2 (0.0–0.8) |
| Chronic obstructive pulmonary disease | 0 | 0.0 (0.0–0.4) | 3 | 0.3 (0.1–0.9) |
| Pulmonary fibrosis | 0 | 0.0 (0.0–0.4) | 2 | 0.2 (0.0–0.8) |

SAE: serious adverse event; pIMD: potential immune-mediated disease; TVC: total vaccinated cohort; RZV: participants receiving the adjuvanted recombinant zoster vaccine; Placebo: participants receiving placebo; CI: confidence interval; ZOE-50/70: RZV efficacy studies in adults ≥50 YOA and ≥70 YOA, respectively; N: number of participants with at least 1 administered dose; n/%: number/percentage of participants reporting the condition at least once; MedDRA: medical dictionary for regulatory activities; SOC: system organ class; PT: preferred term; YOA: years of age.

Per PT, only SAEs reported by ≥0.3% of RZV recipients and SAEs with fatal outcome reported by ≥0.2% in either group are presented here.

**Members of ZOE-50/70 study group:** Andrews, Charles; Brotons Cuixart, Carles; Caso, Covadonga; de Looze, Ferdinandus; Desole, Maria Giuseppina; Diez Domingo, Javier; Eizenberg, Peter; Gorfinkel, Iris; Hwang, Shinn-Jang; Ikematsu, Hideyuki; Leung, Edward; Mascarenas de Los Santos, Abiel; Narejos Perez, Silvia; Pauksens, Karlis; Puig Barbera, Joan; Ribeiro dos Santos, Rodrigo; Rodriguez de la Pinta, Maria Luisa; Rombo, Lars; SMETANA, Jan; Staniscia, Tommaso; Tinoco, Juan Carlos; Toma, Azhar; Gervais, Pierre; Hui, David Shu Cheong; Ahonen, Anitta; Berglund, Johan; Chlibek, Roman; Cunningham, Anthony L; Watanabe, Daisuke; Weckx, Lily; Downey, H. Jackson; Korhonen, Tiina; McNeil, Shelly; Athan, Eugene; Avelino-Silva, Thiago Junqueira; Barba-Gomez, Jose-Fernando; Choi, Won Suk; Esen, Meral; Johnson, Robert W.; McElhaney, Janet; Schwarz, Tino F; Yeo, Wilfred; Pellegrino, Angelo; Poder, Airi; Seppa, Ilkka; Thompson, Alexander**.**
